# Predicting postoperative pulmonary infection risk in patients with diabetes using machine learning

**DOI:** 10.3389/fphys.2024.1501854

**Published:** 2024-12-04

**Authors:** Chunxiu Zhao, Bingbing Xiang, Jie Zhang, Pingliang Yang, Qiaoli Liu, Shun Wang

**Affiliations:** ^1^ Department of Critical Care Medicine, Affiliated Hospital of Southwest Jiaotong University, The Third People's Hospital of Chengdu, Chengdu, Sichuan, China; ^2^ Department of Anesthesiology, West China Hospital, Sichuan University, Chengdu, China; ^3^ Department of Anesthesiology, Clinical Medical College and The First Affiliated Hospital of Chengdu Medical College, Chengdu, Sichuan, China

**Keywords:** diabetes mellitus, postoperative pulmonary infection, machine learning, risk prediction, Ada Boost classifier

## Abstract

**Background:**

Patients with diabetes face an increased risk of postoperative pulmonary infection (PPI). However, precise predictive models specific to this patient group are lacking.

**Objective:**

To develop and validate a machine learning model for predicting PPI risk in patients with diabetes.

**Methods:**

This retrospective study enrolled 1,269 patients with diabetes who underwent elective non-cardiac, non-neurological surgeries at our institution from January 2020 to December 2023. Predictive models were constructed using nine different machine learning algorithms. Feature selection was conducted using Least Absolute Shrinkage and Selection Operator (LASSO) logistic regression. Model performance was assessed via the Area Under the Curve (AUC), precision, accuracy, specificity and F1-score.

**Results:**

The Ada Boost classifier (ADA) model exhibited the best performance with an AUC of 0.901, Accuracy of 0.91, Precision of 0.82, specificity of 0.98, PPV of 0.82, and NPV of 0.82. LASSO feature selection identified six optimal predictive factors: postoperative transfer to the ICU, Age, American Society of Anesthesiologists (ASA) physical status score, chronic obstructive pulmonary disease (COPD) status, surgical department, and duration of surgery.

**Conclusion:**

Our study developed a robust predictive model using six clinical features, offering a valuable tool for clinical decision-making and personalized prevention strategies for PPI in patients with diabetes.

## Introduction

The global prevalence of diabetes is on the rise, with an estimated 783 million individuals expected to be affected by the condition by 2045 (Magliano and Boyko, 2021). Patients with diabetes are at a higher risk of developing complications following surgical procedures compared to patients without diabetes (Crowley et al., 2023). PPI represent one of the most common complications after surgery, leading to increased patient suffering, higher medical costs, and placing a significant burden on public health systems (Jiang et al., 2024; Abbott et al., 2018; Verkoulen et al., 2024). Therefore, effectively predicting and preventing PPI in patients with diabetes has become an urgent issue that needs addressing.

In this context, employing advanced data analysis techniques to identify high-risk patients and implement targeted preventive measures is of critical importance. In recent years, machine learning has emerged as a powerful tool for data mining, finding extensive applications in healthcare, particularly in the development of disease prediction models (Sidey-Gibbons and Sidey-Gibbons, 2019; Deo, 2015). By analyzing large volumes of clinical data, machine learning algorithms can uncover potential risk factors and their interrelationships, aiding in the early identification of high-risk groups and guiding clinical decision-making, thereby reducing the incidence of PPI (Li et al., 2023; NIHR Global Health Research Unit on Global Surgery, STARSurg Collaborative, 2024). While some studies have begun to explore the use of machine learning methods to predict the risk of PPI, there is a relative paucity of research dedicated to developing prediction models specifically for patients with diabetes (Odor et al., 2020; Kouli et al., 2022). Consequently, the development of a prediction model tailored to the risk of PPI in patients with diabetes is of paramount importance.

Against this backdrop, the present study aims to utilize machine learning algorithms to develop a predictive model for the risk of PPI in patients with diabetes. It is hoped that this model will effectively identify those patients who are at high risk of developing PPI, providing clinicians with more personalized prevention and intervention strategies. Additionally, this study will explore the applicability and accuracy of different machine learning algorithms in predicting PPI, offering valuable insights for clinical practice.

Through this research, it is anticipated that new ideas and technological support will be provided for the postoperative management of patients with diabetes. Furthermore, this study will lay the groundwork for future large-scale, multicenter prospective studies, contributing to the advancement of efforts aimed at preventing PPI.

## Methods

### Study design

This study employed a retrospective cross-sectional design. Clinical data were collected from patients with diabetes who underwent elective non-cardiac, non-neurological surgeries at our institution between January 2020 and December 2023.

### Patient and public involvement

Patients or the public WERE NOT involved in the design, or conduct, or reporting, or dissemination plans of our research.

## Data collection

### Inclusion and exclusion criteria

Inclusion criteria were: 1. Aged 18–75 years, regardless of gender. 2. Undergoing elective non-cardiac and non-neurological surgery. 3. Diagnosed with Type II diabetes. Exclusion criteria were: 1. Preoperative pulmonary infection. 2. Body mass index (BMI) ≥ 35 kg/m^2^ 3. Significant abnormalities in cardiac, pulmonary, hepatic, or renal function. 4. Physical status classification ≥ IV. 5. Missing Hemoglobin A1c (HbA1c) data. 6. Excessive missing data.

### Data sources and preprocessing

Relevant patient information was extracted from the hospital’s electronic medical record system. Specific data sources included: Inpatient medical records; Surgical reports; Anesthesia records; Laboratory test reports; Imaging examination reports; Nursing records.

The collected data primarily encompassed: 1. Demographic characteristics: Age, gender, height, weight, etc; 2. Diabetes-related information: Medication adherence, insulin therapy usage; 3. Comorbidities: Hypertension, coronary heart disease, COPD; 4. Laboratory tests: HbA1c, average blood glucose levels within 3 days postoperatively; 5. Surgical information: Surgical department, duration of surgery, type of anesthesia; 6. Preoperative assessment: ASA classification, New York Heart Association (NYHA) classification, preoperative oral carbohydrate, baseline blood pressure, baseline heart rate; 7. Postoperative management: Use of PCA, transfer to the ICU after surgery; 8. Other: Blood transfusion during perioperative period.

### Data preprocessing

Data Cleaning: 1. Identification and correction of obvious data entry errors; 2. Handling of duplicate records.

Missing Value Handling: 1. For variables with a missing rate <5%, median or mean imputation was applied; 2. For variables with a missing rate between 5% and 10%, the decision to impute or exclude was based on the variable’s importance and the pattern of missingness; 3. Variables with a missing rate >10% were excluded.

Outlier Handling: All outlier data points were removed to minimize their impact on the analysis.

Feature Engineering: 1. Standardization of continuous variables: Z-score standardization method was used; 2. Encoding of categorical variables: One-hot encoding was applied; 3. Creation of composite features: BMI based on height and weight, Mean Arterial Pressure (MAP) derived from systolic and diastolic blood pressures.

### Main outcomes

The primary outcome of this study was the incidence of pulmonary infections diagnosed by the operating surgeon before patient discharge. PPI were defined as new-onset pulmonary infections diagnosed postoperatively.

### Model selection and training

We selected nine commonly used machine learning algorithms to build our predictive models: K-Nearest Neighbors (KNN): An instance-based learning algorithm. Support Vector Machine (SVM) with Linear Kernel: A supervised learning algorithm for classification. Random Forest (RF): An ensemble learning method based on decision trees. Decision Tree (DT): A tree-based classification algorithm. Light Gradient Boosting Machine (LightGBM): A gradient boosting framework. Ada Boost (ADA): An ensemble boosting algorithm. Naive Bayes (NB): A probabilistic classifier based on Bayes’ theorem. Logistic Regression (LR): A statistical method for binary outcome prediction. Linear Discriminant Analysis (LDA): A method for finding linear combinations of features that separate classes.

Then, LASSO logistic regression was used to select PPI features (Mullah et al., 2021). The dataset was randomly divided into a training set and a testing set at a ratio of 3:1. The model was trained and optimized using 10-fold cross-validation on the training set.

### Model evaluation

The performance of the models was evaluated using the following metrics: 1. AUC; 2. Accuracy; 3. Precision; 4. Specificity; 5. PPV; 6. NPV.

To enhance the interpretability of the models, SHAP (SHapley Additive exPlanations) values were utilized to analyze the contribution of each feature to the prediction results.

### Preventive strategy formulation

Based on the predictions from the machine learning models and the analysis of feature importance, we aimed to: 1. Identify high-risk patient groups. 2. Quantify the impact of each risk factor. 3. Develop targeted preventive measures and intervention plans.

### Statistical analysis

Continuous variables were summarized using means ± standard deviation (SD) for normally distributed data or medians with interquartile ranges (IQR) for non-normally distributed data. Categorical variables were described using frequencies and percentages. Machine learning models were built and evaluated using R version 4.2.2 with the caret package and the pROC package. A *p*-value <0.05 was considered statistically significant.

### Ethics approval and consent to participate

This study was approved by the Ethics Committee of the First Affiliated Hospital of Chengdu Medical College (2023CYFYIRB-BA-May03) and registered in the Chinese Clinical Trial Registry (ChiCTR2400080801, www.chictr.org.cn). The requirement for informed consent was waived due to the retrospective nature of the study. All procedures were conducted in accordance with the Declaration of Helsinki and relevant institutional guidelines (von et al., 2007).

## Study results

### Baseline characteristics of study participants

**FIGURE 1:**
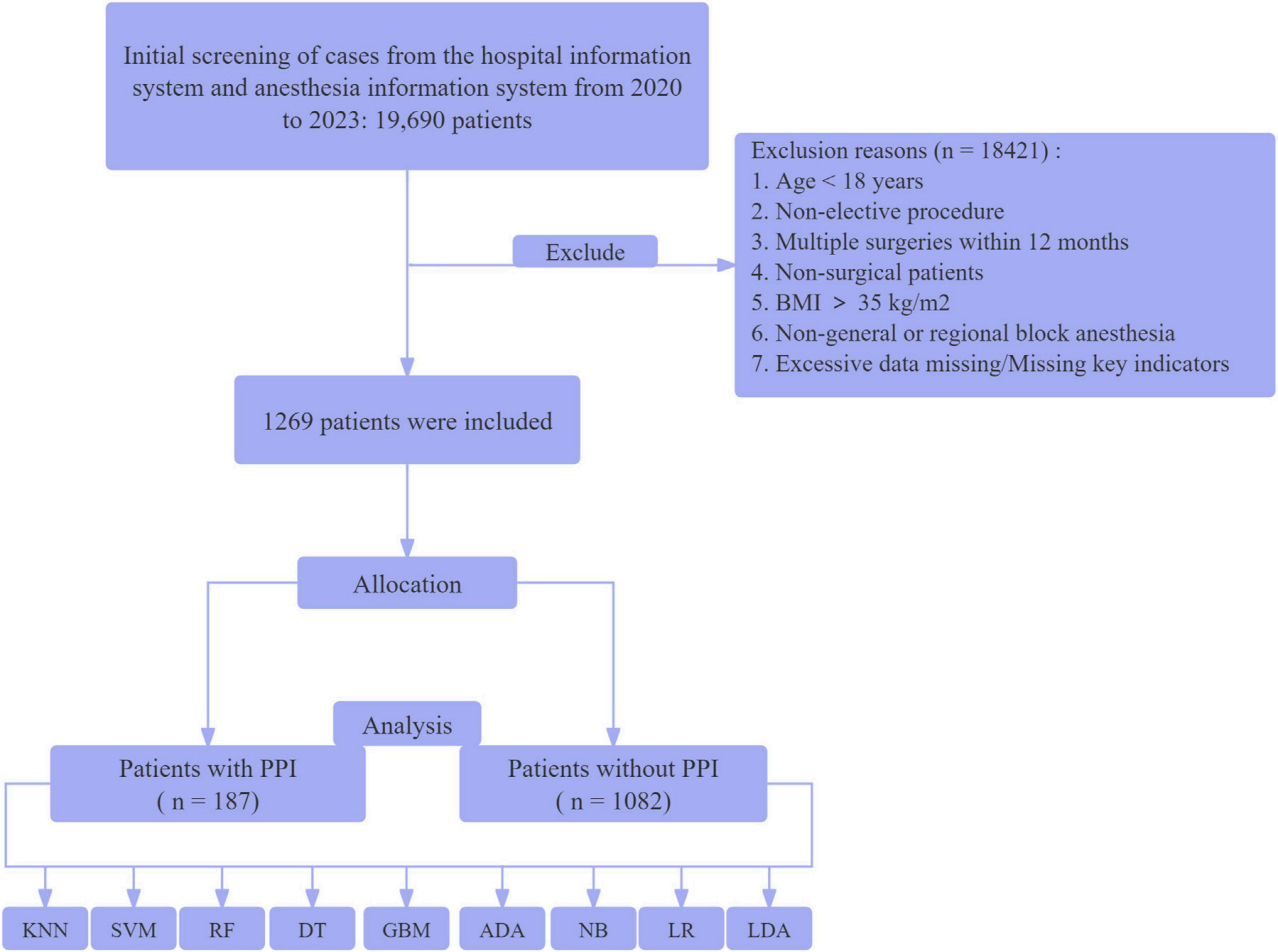
Flow diagram of the study selection process. K-Nearest Neighbors Classifier (KNN), Support Vector Machine with Linear Kernel (SVM - Linear Kernel), Random Forest Classifier (RF), Decision Tree Classifier (DT), Light Gradient Boosting Machine (Light GBM), Ada Boost Classifier (ADA), Naive Bayes (NB), Logistic Regression (LR), and Linear Discriminant Analysis (LDA).

Between January 2020 and December 2023, a total of 19,690 patients with diabetes underwent surgical treatment at our institution. After screening, 1,269 patients were ultimately included for analysis. The patient enrollment flowchart is shown in Figure 1, and the baseline characteristics are presented in Table 1.

**TABLE 1:** Patients group baseline characteristics by primary outcomes.

Variable	Patients with PPI (n = 187)	Patients without PPI (n = 1,082)	P
Sex (Female, n, %)	80 (42.78%)	621 (57.39%)	<0.001
Age (years)	64.24 (8.37)	60.43 (9.96)	<0.001
Hight (cm)	159.88 (7.43)	159.19 (7.78)	0.260
Weight (kg)	61.65 (11.69)	63.07 (11.24)	0.112
BMI (kg/m2)	24.07 (4.02)	24.84 (3.77)	0.011
CHO (n, %)	45 (24.06%)	179 (16.54%)	0.013
Departments			<0.001
Department of Orthopedic Surgery (n, %)	34 (18.18%)	229 (21.16%)	
Department of Gastrointestinal Surgery (n, %)	27 (14.44%)	58 (5.36%)	
Department of Thoracic and Cardiovascular Surgery (n, %)	83 (44.39%)	31 (2.87%)	
Other Departments	43 (22.99%)	764 (70.61%)	
PCA (n, %)	144 (77.01%)	587 (54.25%)	<0.001
ASA (n, %)			<0.001
II	83 (44.39%)	745 (68.85%)	
III	104 (55.61%)	337 (31.15%)	
Perioperative blood transfusion (n, %)	27 (14.43%)	52 (4.81%)	
Basic SBP (mmHg)	135.56 (17.86)	137.13 (18.39)	0.281
Basic DBP (mmHg)	76.10 (11.02)	77.24 (11.51)	0.208
Basic MAP (mmHg)	95.92 (11.17)	97.21 (12.02)	0.171
Basic HR (bpm)	83.19 (12.76)	83.10 (12.15)	0.931
HbA1c (%)	7.60 (2.6)	7.45 (2.8)	0.604
Hypertension (n, %)	103 (55.08%)	507 (46.86%)	0.038
CHD (n, %)	20 (10.70%)	75 (6.93%)	0.071
COPD (n, %)	18 (9.62%)	31 (2.87%)	<0.001
NYNA (n, %)			<0.001
1	110 (58.82%)	800 (73.94%)	
2	68 (36.36%)	262 (24.21%)	
3	9 (4.81%)	20 (1.85%)	
Transfer to ICU (n, %)	21 (11.23%)	16 (1.48%)	<0.001
Diabetes drug (n, %)	138 (73.80%)	740 (68.39%)	0.139
Insulin (n, %)	22 (11.76%)	94 (8.69%)	0.178
Mean blood glucose on Pos - 1 d (mmol/L)	11.20 (3.5)	11.40 (3.4)	0.314
Mean blood glucose on Pos - 2 d (mmol/L)	8.3 (2.8)	8.4 (2.8)	0.525
Mean blood glucose on Pos - 2 d (mmol/L)	7.9 (2.8)	7.9 (2.8)	0.594
Blood glucose variability (%)	20.20 (13.10)	22.20 (13.35)	0.110
Surgery time (min)	359.90 (171.12)	237.86 (125.61)	<0.001
Postoperative hospital stay (days)	9.70 (7.6)	6.15 (5.0)	<0.001
General anesthesia (n, %)	176 (94.12%)	877 (81.05%)	<0.001

*Note:* Data are expressed as M (IQR) or number of patients (%) as appropriated. PPI, postoperative pulmonary infection; ICU, intensive care unit; PCA, Patient-Controlled Analgesia; IQR, interquartile range; BMI, body mass index; MAP, mean arterial pressure; HbA1c, Hemoglobin A1c; NYHA, new york heart association; ASA, american society of anesthesiologists; COPD, chronic obstructive pulmonary disease; SBP, systolic blood pressure; DBP, diastolic blood pressure; HR, heart rate.

Of the 1,269 patients included in the study, 187 (14.7%) developed PPI. Patients who developed PPI were significantly older (64.24 ± 8.37 vs. 60.43 ± 9.96 years, *P* < 0.001), had a higher ASA score (*P* < 0.001), and were more likely to have COPD (9.62% vs. 2.87%, *P* < 0.001) compared to those who did not develop PPI. The duration of surgery was also significantly longer in the PPI group (359.90 ± 171.12 vs. 237.86 ± 125.61 min, *P* < 0.001).

### Feature selection using LASSO - Logistic regression method

We used LASSO (Least Absolute Shrinkage and Selection Operator) logistic regression for feature selection, implemented using the glmnet package in R. The optimal regularization parameter λ was selected through cross-validation using cv. glmnet function. Feature importance was ranked based on the absolute values of their non-zero coefficients.

**FIGURE 2:**
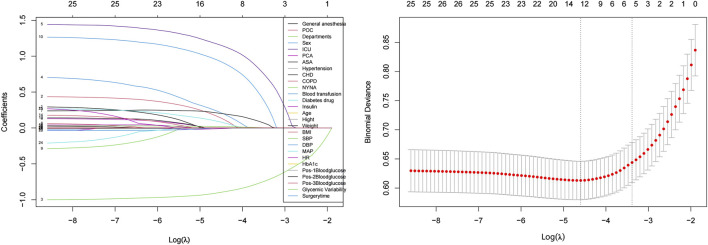
Feature selection using the LASSO-logistic regression method. Left panel shows LASSO coefficient profiles of the 28 features versus log(λ). Numbers at the top represent the count of nonzero coefficients. Right panel shows cross-validated error (binomial deviance) versus log(λ). Two vertical dotted lines represent λ.min (left) and λ.1se (right). At λ.1se, six features were selected: postoperative ICU admission, age, ASA classification, COPD status, surgical department, and duration of surgery.

Due to the potential negative impact of correlated or less important features on the performance of machine learning models (Figure 2), we conducted feature selection and ranked the importance of the features.

### Optimal features

Using the LASSO-Logistic feature selection method, we identified 12 optimal features from the initial 28. These features performed exceptionally well in predicting the risk of PPI in patients with diabetes: Sex; Postoperative ICU admission; MAP; Systolic Blood Pressure (SBP); ASA classification; COPD; NYHA classification; BMI; Preoperative oral carbohydrate loading; Surgical departments; Age; Duration of surgery.

### Recommended features

To further simplify the model and enhance its interpretability, we selected six of the most important features. These features exhibit the highest importance in predicting PPI: Postoperative ICU admission; Age; ASA classification; COPD; Surgical department; Duration of surgery.

Using these recommended features for model training not only ensures model performance but also simplifies the model structure, enhancing its interpretability. These results provide strong support for subsequent clinical decision-making and the development of personalized preventive strategies.

### Feature importance display

**FIGURE 3:**
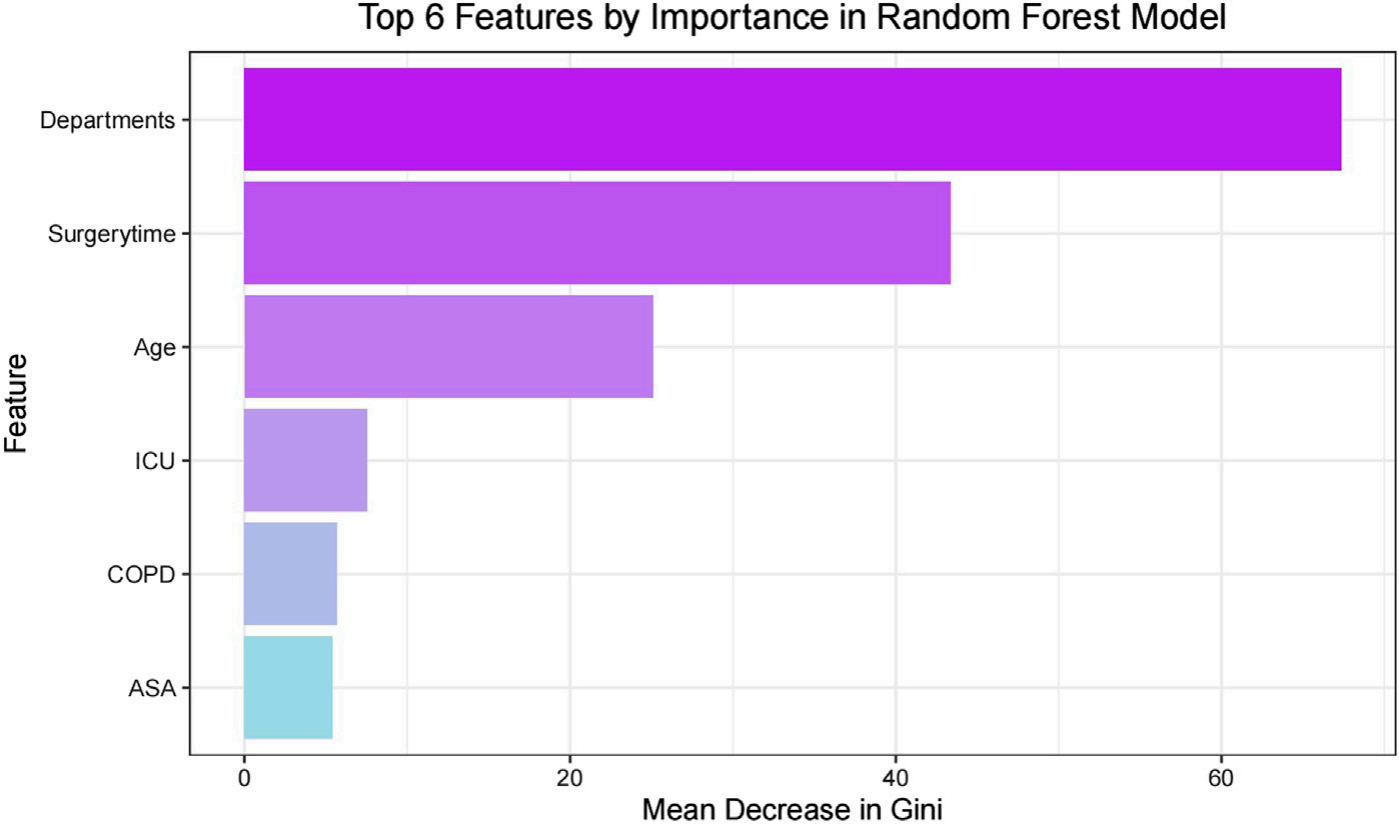
Top features by importance in random forest model.

Random Forest analysis of the six selected features revealed their relative importance through Mean Decrease in Gini index (Figure 3). Surgical department emerged as the most influential predictor, followed by surgery duration and age, while ICU admission, COPD status, and ASA classification showed relatively lower contributions. This ranking suggests that surgical type and procedural factors have stronger predictive power than patient baseline characteristics.

### Performance metrics of each model on the train and test set

**FIGURE 4:**
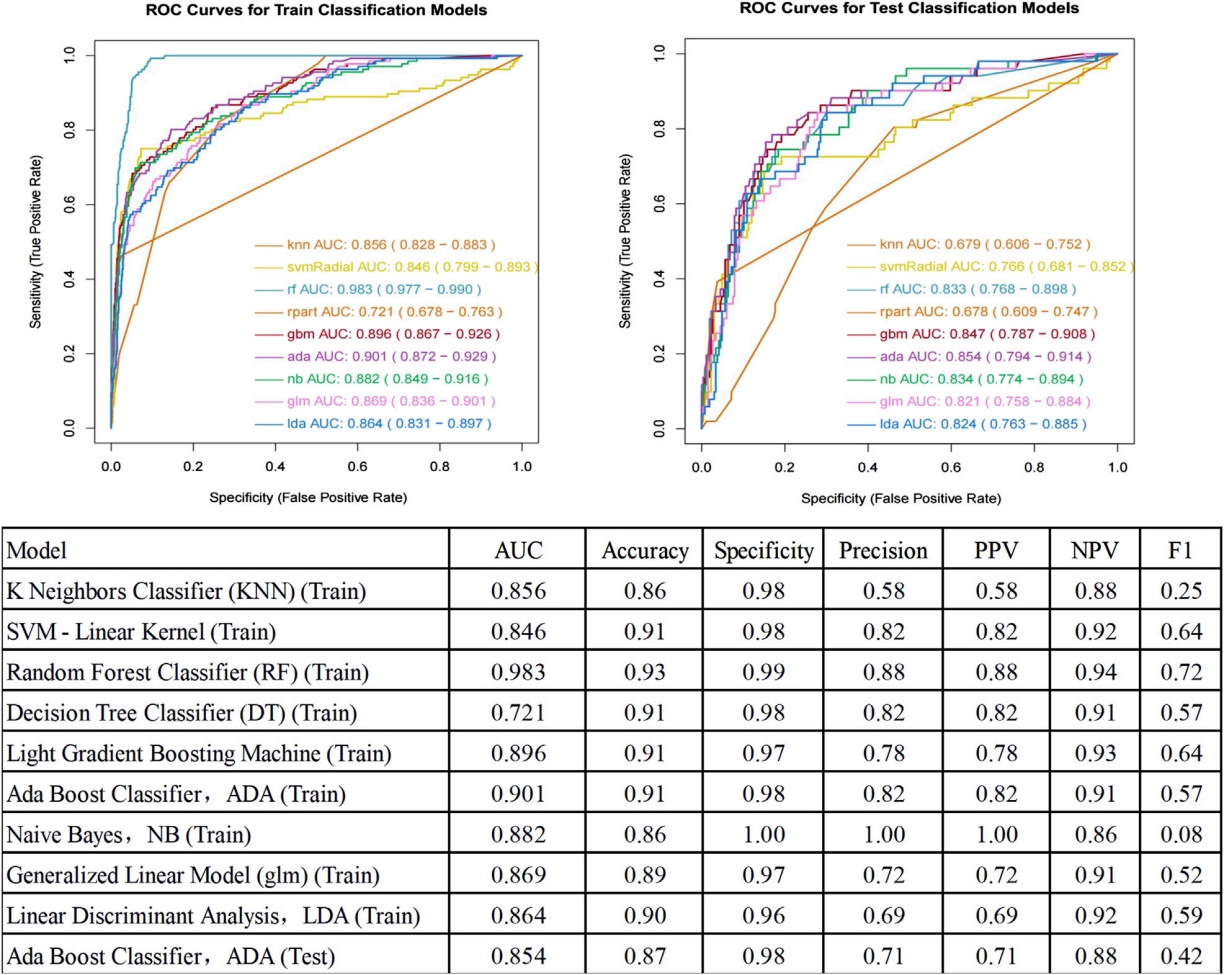
ROC curve for prediction of postoperative pulmonary infection and Performance Metrics of Each Model on the Test Set. AUC, Area Under the Curve; ROC, Receiver Operating Characteristic; PPV, Positive Predictive Value; NPV, Negative Predictive Value.

The performance metrics of each model on the train and test set are summarized in Figure 4. The Ada Boost (ADA) model performed the best across all metrics, with its ROC curve illustrated in Figure 4.

### Key predictors and their clinical implications

Our LASSO-Logistic model identified six critical predictors of PPI risk in diabetic patients: 1. Surgical Department: Thoracic surgery carried the highest risk (72.81% vs 5.33% in other departments, P<0.001), followed by gastrointestinal surgery (31.76%) and orthopedics (12.93%), likely due to direct respiratory system involvement and procedure complexity. 2. Postoperative ICU Admission: Strongly indicative of case complexity and patient vulnerability (11.23% in PPI group vs 1.48% in non-PPI group, P<0.001). These patients typically require more intensive monitoring and have higher severity of underlying conditions. 3. Age: Significantly higher in PPI group (64.24 ± 8.37 vs 60.43 ± 9.96 years, P<0.001), associated with immunosenescence, pulmonary function decline, reduced recovery capacity, and increased comorbidities. Advanced age particularly impacts respiratory defense mechanisms and wound healing. 4. ASA Classification: Higher proportion of Grade III in PPI group (55.61% vs 31.15%, P<0.001), reflecting poorer overall health status, multiple systemic comorbidities, and increased complication susceptibility. 5. COPD Status: Markedly higher prevalence in PPI group (9.62% vs 2.87%, P<0.001), significantly elevating PPI risk due to impaired lung function, compromised respiratory defense mechanisms, and chronic airway inflammation. 6. Surgery Duration: Substantially longer in PPI group (359.90 ± 171.12 vs 237.86 ± 125.61 min, P<0.001). Longer procedures increase pathogen exposure, tissue trauma, anesthesia-related risks, and physiological stress response.

To ensure data consistency and representativeness, we analyzed the distribution of patient characteristics across different surgical departments and risk groups. The 1,269 patients were distributed across thoracic surgery (8.98%, n = 114), orthopedics (20.72%, n = 263), gastrointestinal surgery (6.70%, n = 85), and other departments (63.60%, n = 807). The incidence of PPI varied significantly among departments: thoracic surgery (72.81%), orthopedics (12.93%), gastrointestinal surgery (31.76%), and other departments (5.33%). This variation reflects the inherent risks associated with different surgical procedures and was successfully captured by our model, with the surgical department being identified as the most important predictor (Figure 3). Key clinical characteristics also showed consistent patterns between PPI and non-PPI groups. Age (64.24 ± 8.37 vs 60.43 ± 9.96 years, P<0.001), ASA classification (Grade III: 55.61% vs 31.15%, P<0.001), and surgery duration (359.90 ± 171.12 vs 237.86 ± 125.61 min, P<0.001) were significantly different between groups, indicating robust discriminative features for our prediction model.

These findings offer valuable insights for clinical practice: 1. Risk Stratification: Enables targeted preventive measures for high-risk patients. 2. Age-Adapted Care: Tailored strategies including enhanced respiratory care, early mobilization, and optimized medication and nutrition for older patients. 3. Preoperative Optimization: Thorough evaluation and management, especially for patients with high ASA scores or COPD. 4. Surgical Planning: Where clinically appropriate, consider minimizing surgery duration and optimizing technique. 5. Specialized Protocols: Implement enhanced postoperative care and monitoring, particularly for thoracic surgery patients.

## Discussion

This study aimed to develop a machine learning-based predictive model for assessing the risk of PPI in patients with diabetes. Our model showed stable performance between training (AUC 0.901) and testing (AUC 0.854). The six predictors identified are widely available clinical parameters, suggesting potential broad applicability. This finding is consistent with recent studies, which have shown that ensemble learning methods, such as Ada Boost, excel in predicting postoperative complications (Koenen et al., 2024; Wu et al., 2023).

Most prior studies (Kouli et al., 2022) have focused on general surgical populations, overlooking the unique risk factors in diabetic patients, leading to suboptimal risk assessment. By targeting diabetic patients, our study provides a more nuanced understanding of risk factors in this high-risk group.

Our ADA Boost model (AUC 0.854, specificity 0.98, accuracy 0.87) showed improved performance compared to recent studies: Li et al.'s (Li et al., 2023) Random Forest model (AUC 0.721, accuracy 0.664, specificity 0.656)) for spinal cord injury patients, Jiang et al. (2024) study of esophageal cancer patients, where they reported model performance ranges of AUC 0.627–0.850, sensitivity 60.7%–84.0%, and specificity 59.1%–83.9%, and Wang et al.'s (Wang et al., 2024) nomogram (AUC 0.759) for post-abdominal surgery ICU patients. This improvement likely results from our model’s ability to capture complex relationships between risk factors and the inclusion of diabetes-specific features, though external validation studies are still needed. Using the LASSO-logistic feature selection method, we identified six optimal predictors: transfer to ICU after surgery, Age, ASA grade, COPD, surgical departments, and surgery time. The identification of these factors not only enhances the predictive accuracy of the model but also provides clear targets for clinical intervention. Particularly, the duration of surgery, being a modifiable factor, is closely associated with surgical trauma and anesthesia time, all of which significantly impact postoperative outcomes. This importance has been substantiated in multiple studies (Gupta et al., 2011; Short et al., 2017; Mehaffey et al., 2017; Seykora et al., 2019; Alrefaei et al., 2024).

Our study results indicate that COPD is one of the key factors in predicting PPI in patients with diabetes. This finding is consistent with several recent studies, underscoring the importance of COPD in the assessment of postoperative complication risks (Shin et al., 2017; Roy et al., 2020; Agostini et al., 2010; Quan et al., 2022; Deng et al., 2024). The significant increase in risk highlights the necessity for special attention to patients with COPD. The association between COPD and PPIs may stem from multiple mechanisms (Vestbo et al., 2013). Firstly, patients with COPD often experience chronic airway inflammation and impaired mucociliary clearance, which increases the risk of bacterial colonization and infection. Secondly, COPD is frequently accompanied by decreased lung function, leading to reduced tolerance to surgical stress and anesthesia (Maddah and Barzegari, 2023; Duggappa et al., 2015). Moreover, the coexistence of COPD and diabetes may further exacerbate the risk of PPIs. This could be due to the interaction between the immune dysfunction caused by diabetes and the structural and functional changes in the lungs induced by COPD (Zhou et al., 2024).

There are significant differences in the incidence of PPI across different surgical departments, with patients undergoing thoracic, orthopedic, and gastrointestinal surgeries being at higher risk. Unsurprisingly, due to the specific nature of the surgical site, patients who undergo thoracic surgery have a notably high rate of PPI (Ailawadi et al., 2017; Poelaert et al., 2014; Alrefaei et al., 2024). Previous studies have also shown relatively high rates of PPI in patients undergoing orthopedic (Song et al., 2016) and gastrointestinal surgeries (Miki et al., 2016), with advanced age and COPD being significantly associated with an increased risk of PPI (Alrefaei et al., 2024). This finding suggests the need for specific preventive strategies tailored to different departments.

Postoperative transfer to the ICU typically indicates that the patient has undergone more complex surgery or has more severe underlying conditions, naturally increasing the risk of PPI (Wang et al., 2024).

For clinical implementation, we propose integrating this model into electronic medical record systems to automatically calculate PPI risk using the six predictors. This enables risk stratification (high/moderate/low) to guide preventive interventions: enhanced measures for high-risk patients, increased monitoring for moderate-risk patients, and standard care for low-risk patients. Such stratification allows for more targeted preventive strategies and efficient resource allocation.

### Strength and limitations and future directions

This study demonstrates significant methodological and clinical strengths by rigorously comparing nine advanced machine learning algorithms and using a large, diverse sample across multiple surgical specialties. LASSO feature selection and SHAP value analysis improve model efficiency and interpretability. Translating findings into actionable preventive strategies bridges the gap between advanced analytics and clinical practice, highlighting the study’s innovative approach and practical relevance to postoperative care for diabetic patients.

However, our study has several limitations. First, this is a retrospective single-center study without external validation. Our patient population and institutional-specific practice patterns (such as surgical protocols and diagnostic criteria) may affect model generalizability. Second, the model’s performance in real-world clinical settings remains untested. Its applicability may vary across different healthcare settings, geographic regions, and institutions. These limitations underscore the need for future external validation studies. Future research should focus on: (1) external validation through multi-center studies, (2) prospective evaluation of clinical effectiveness, and (3) assessment of model performance in different healthcare settings. These steps are crucial for validating the model’s clinical utility (Ramspek et al., 2021).

## Conclusion

This study presents an accurate and interpretable machine learning model for predicting PPI risk in persons with diabetes. By identifying key risk factors, the model aids in clinical decision-making and personalized preventive strategies, potentially improving outcomes and resource allocation. While our study provides a robust foundation for predicting PPI risk in diabetic surgical patients, future research should focus on external validation, model refinement, dynamic risk prediction, intervention studies, comparative effectiveness research, and economic analysis.

## Data Availability

The raw data supporting the conclusions of this article will be made available by the authors, without undue reservation.

## Abbreviations

PPI, Postoperative Pulmonary Infection; ICU, Intensive Care Unit; PCA, Patient-Controlled Analgesia; ASA, American Society of Anesthesiologists; COPD, Chronic Obstructive Pulmonary Disease; NYHA, New York Heart Association; LASSO, Least Absolute Shrinkage and Selection Operator; AUC, Area Under the Curve; ROC, Receiver Operating Characteristic; PPV, Positive Predictive Value; NPV, Negative Predictive Value; KNN, K-Nearest Neighbors; SVM, Support Vector Machine; RF, Random Forest; DT, Decision Tree; LightGBM, Light Gradient Boosting Machine; ADA, Ada Boost; NB, Naive Bayes; LR, Logistic Regression; LDA, Linear Discriminant Analysis; SHAP, SHapley Additive exPlanations; SD, Standard Deviation; IQR, Interquartile Range; BMI, Body Mass Index; MAP, Mean Arterial Pressure; HbA1c, Hemoglobin A1c; SBP, Systolic blood pressure; DBP, Diastolic blood pressure; HR, Heart rate.
